# Evaluating the use of eye tracking tasks as language‐ and culturally‐neutral assessments of cognition in a multi‐ethnic cohort of older adults

**DOI:** 10.1002/alz.090577

**Published:** 2025-01-09

**Authors:** Rachel Yep, Alexander J Nyman, Georgia Gopinath, Madeline Wood Alexander, Tulip Marawi, Silina Z Boshmaf, Donald C Brien, Brian C Coe, Christopher B Pople, Douglas P Munoz, Jennifer D Ryan, Sandra E Black, Maged Goubran, Jennifer S Rabin

**Affiliations:** ^1^ Sunnybrook Research Institute, Toronto, ON Canada; ^2^ University of Toronto, Toronto, ON Canada; ^3^ Queen's University, Kingston, ON Canada; ^4^ Rotman Research Institute, Toronto, ON Canada

## Abstract

**Background:**

Canada and the United States are both aging and becoming increasingly diverse. Despite this demographic shift, non‐White racial/ethnic groups remain underrepresented in research on cognitive impairment and dementia. A major barrier to inclusivity is the lack of cognitive assessments that are valid in individuals with diverse language and cultural backgrounds. Eye tracking tasks can potentially overcome this barrier since they do not require verbal responses and use culturally neutral stimuli. Here, we tested this hypothesis by comparing performance on standard neuropsychological tests and eye tracking tasks across individuals from White and underrepresented ethnic groups (UEG).

**Method:**

Participants were cognitively unimpaired adults enrolled in the Canadian Multi‐Ethnic Research on Aging (CAMERA) study. Participants completed a battery of standard neuropsychological tests and two well‐characterized eye tracking tasks: the interleaved pro/anti‐saccade task (IPAST), which assesses executive function, and the visual paired comparison task (VPCT), which assesses visual memory.

**Result:**

We included 54 participants (mean age=68.68±6.91, 70% female) with a Clinical Dementia Rating global score of 0. Among them, 22% of participants self‐identified as White (n=12) and the remaining participants identified as being from an UEG (n=42; 37% East Asian, 37% South Asian, 4% Other). Linear regression models assessed whether ethnic background (White vs. UEG) was associated with task performance after adjusting for age, sex, and years of education. We found that ethnic background influenced performance on several standard neuropsychological tests, including the MoCA (β=0.26, p=0.03), phonemic fluency (β=0.29, p=0.02), category fluency (β=0.38, p=0.003), and Benson Complex Figure delay (β=0.23, p=0.08), such that the UEG group had lower scores. There were no group differences on Trails A and B or the Symbol Digit Modalities Test (p‐values>0.1). Importantly, ethnic background did not influence performance on either eye tracking task (IPAST: β=‐0.17, p=0.17; VPCT: β=0.02, p=0.83).

**Conclusion:**

Our preliminary results suggest that ethnic background influences performance on several standard neuropsychological tests, but not performance on the IPAST or VPCT. With further validation in larger samples, these eye tracking tasks may provide a brief and language‐free way to screen and monitor cognitive impairment in diverse samples currently underrepresented in dementia research.

